# Thermal Effects on Domain Wall Stability at Magnetic Stepped Nanowire for Nanodevices Storage

**DOI:** 10.3390/nano14141202

**Published:** 2024-07-15

**Authors:** Mohammed Al Bahri, Salim Al-Kamiyani

**Affiliations:** Department of Basic and Applied Sciences, A’Sharqiyah University, P.O. Box 42, Ibra P.C 400, Oman; salim.alkamiyani@asu.edu.om

**Keywords:** micromagnetic simulation, DW thermal stability, magnetic domain wall, stepped magnetic nanowire, spin transfer torque

## Abstract

In the future, DW memory will replace conventional storage memories with high storage capacity and fast read/write speeds. The only failure in DW memory arises from DW thermal fluctuations at pinning sites. This work examines, through calculations, the parameters that might help control DW thermal stability at the pinning sites. It is proposed to design a new scheme using a stepped area of a certain depth (*d*) and length (*λ*). The study reveals that DW thermal stability is highly dependent on the geometry of the pinning area (*d* and λ), magnetic properties such as saturation magnetization (*Ms*) and magnetic anisotropy energy (*Ku*), and the dimensions of the nanowires. For certain values of *d* and *λ*, DWs remain stable at temperatures over 500 K, which is beneficial for memory applications. Higher DW thermal stability is also achieved by decreasing nanowire thickness to less than 10 nm, making DW memories stable below 800 K. Finally, our results help to construct DW memory nanodevices with nanodimensions less than a 40 nm width and less than a 10 nm thickness with high DW thermal stability.

## 1. Introduction

**Figure 1:**
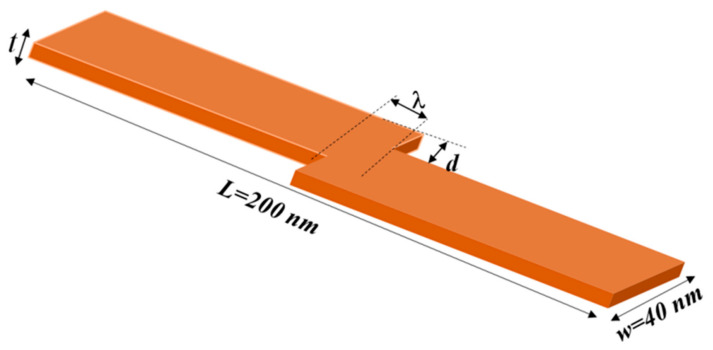
A magnetic nanodevice with a stepped junction at its center that has sizes of depth (*d*) and length (*λ*).

During the past few years, ferromagnetic nanodevices and their applications, such as memories, have attracted extensive research [1,2,3,4]. Recent research in domain wall memory and spintronics explores materials like insulating ferrimagnetic garnets, which show promise for faster domain wall motion with reduced energy dissipation. Additionally, research on racetrack memory, a type of domain wall memory (DWM), aims to improve data density and access speeds by utilizing three-dimensional configurations [5,6,7]. These DWM devices promise non-volatile, high-density, fast data access with low power consumption [8,9,10,11,12,13]. To achieve these properties, DWMs are constructed with nanoscale dimensions and include pinning sites (e.g., notches) to define magnetic states for data storage [14,15,16,17,18]. However, one parameter that reduces domain wall stability at the pinning sites and leads to DWM failure is device temperature. Various studies have attempted to address this issue in different ways. Experimentally, some researchers fabricate devices with microscale dimensions or thin films to reduce temperature effects [19,20,21,22]. Other studies drive the domain walls in magnetic nanowires using magnetic fields to prevent temperature rises in devices [23,24,25,26,27]. For new spintronics applications, such as racetrack memory, spin-transfer torque is more effective than magnetic fields for driving domain walls in magnetic nanowires. With spin-transfer torque, DW velocity increases linearly with current density, allowing for a low enough current density to achieve high storage speeds [28,29]. Additionally, spin-transfer torque allows for more accurate control of DW motion and pinning along magnetic nanowires [1,30]. Theoretically, simulation studies have been conducted in a perfect atmosphere at 0 K to avoid any magnetization perturbation due to device temperature [16,31,32,33,34]. Therefore, in our study, we aim to investigate the DW pinning strength due to device temperature in magnetically stepped nanowires. The stepped nanowire is implemented by creating an offset at the center area by shifting one part in one or two directions, as shown in Figure 1. This design offers benefits such as high pinning strength by altering pinning area dimensions, DW pinning at the stepped area within tens of nanometers, and low-cost fabrication. Previous studies have examined pinning and depinning in stepped devices theoretically at 0 K [35,36,37]. However, none have focused on thermal stability in the stepped area, a crucial parameter affecting DW depinning. Thus, our work focuses on the effects of device temperature on DW depinning in the constricted area at nanoscales. We study the effects of parameters such as magnetic properties, stepped area dimensions, and nanowire dimensions on thermal fluctuations. To implement this study, we perform calculations to examine these parameters, which can be used to control the thermal DW stability at the pinning sites. Initially, we explore the influence of the stepped area’s dimensions on DW thermal stability. We began by varying the depth and length of the stepped area to determine their effect on the DW depinning temperature. It was observed that increasing the stepped area depth increases the DW depinning temperature, indicating higher thermal stability, while increasing the length reduces it. Next, we studied the effect of varying the nanowire thickness on DW thermal stability. The results indicate that thicker nanowires improve DW thermal stability. Then, we explored the impact of magnetic properties such as *Ms* and *Ku* on DW thermal stability in the constricted area. The results show that enhancing *Ms* or *Ku* values leads to increased DW thermal stability. Lastly, we analyzed the thermal stability factor and the effect of device temperature on DW retention in the pinning area. The findings indicate that reducing the device temperature enhances the thermal stability factor, thereby extending the memory lifetime.

## 2. Theoretical Model

In order to investigate the influences of thermal fluctuations on DW depinning in the stepped nanowire, a micromagnetic simulation was performed with the OOMMF software [38]. Landau–Lifshitz–Gilbert (LLG) equation was used with thermal field and spin-transfer torque terms (STTs) in the simulations.

(1) $$\frac{dm}{dt}=-\gamma m\times H_{eff}+H_{th}+\alpha m\times \frac{dm}{dt}-(u\cdot \nabla )m+\beta m\times (u\cdot \nabla )m$$

***m*** represents the magnetization vector, *γ* represents the gyromagnetic ratio, *α* represents the Gilbert damping constant, *H_th_* represents the thermal field, and the vector ***u*** represents the adiabatic spin torque with the velocity dimension [39]. For materials with in-plane magnetic anisotropy like Ni_81_Fe_19_ and CO_90_Fe_10_, parameters were selected. In our simulation, we set the damping coefficient, *α*, to 0.01, the nonadiabatic β to 0.04, and *A* = 2 × 10^−11^ J/m.

The dependence of the thermal field (*H_th_*) on the device’s temperature (*T*) follows the following relation:

(2) $$\langle H_{th,i}\left(\vec{r},t\right),H_{th,j}(\vec{\overset{´}{r},}\overset{´}{t}\rangle =\frac{2\alpha k_{B}T}{\gamma \mu_{0}M_{s}V}\delta_{ij}\delta (\vec{r}-\vec{\overset{´}{r}})\delta (t-\overset{´}{t})$$

This equation uses $k_{B}$ to represent the Boltzmann constant, and *T* to denote the temperature; $\mu_{0}$ and *V* stand for the vacuum permeability and the characterization volume of the cell, respectively [32].

Magnetic nanowires with stepped junctions were designed in this study to have a length (*L*), width (*W*), and thickness (*t*) of 200 × 40 × 3 nm^3^, respectively, as shown in Figure 1. The cell size was chosen to be 2.5 × 2.5 × 3 nm^3^, which is notably smaller than the exchange length and Gilbert damping parameter. Shifting one of the parts vertically (*d*) and horizontally (*λ*) creates a stepped junction at the nanowire’s center to trap the DW [Figure 1].

## 3. Results and Discussion

**Figure 2:**
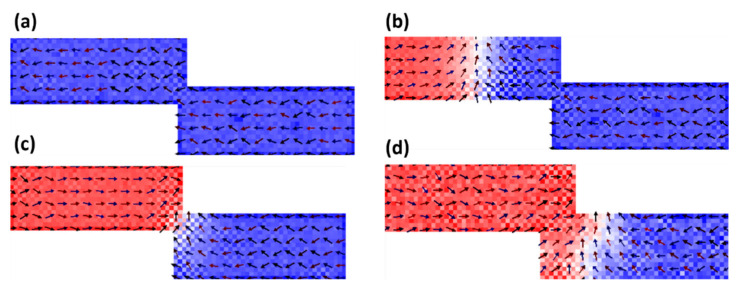
(**a**) The initial state showing nanowire magnetization saturated in the negative *x*-direction. (**b**) Movement of the DW towards the stepped area. (**c**) The DW pinned at the stepped junction under a current density of *J* = 6.63 × 10^12^ Am^−2^ and temperature T = 50 K. (**d**) DW depinning at a temperature of 300 K The nanowire’s stepped junction has dimensions of 30 nm in depth and 5 nm in length.

The magnetization was first saturated by directing the current density in the negative *x*-direction, as depicted in Figure 2a. Next, the current density was switched to the positive *x*-direction to create a DW from the left edge of the nanowire. The DW accelerates under this current towards the stepped area, where it is pinned [Figure 2b,c]. The temperature of the device is then increased until the DW depins and moves to the end of the nanowire [Figure 2d].

The thermal stability of domain walls (DWs) in stepped nanowires was studied by considering various parameters, including the dimensions of the stepped area, the magnetic properties, the nanowire dimensions, and the current density.

### 3.1. Stepped Area Dimensions

**Figure 3:**
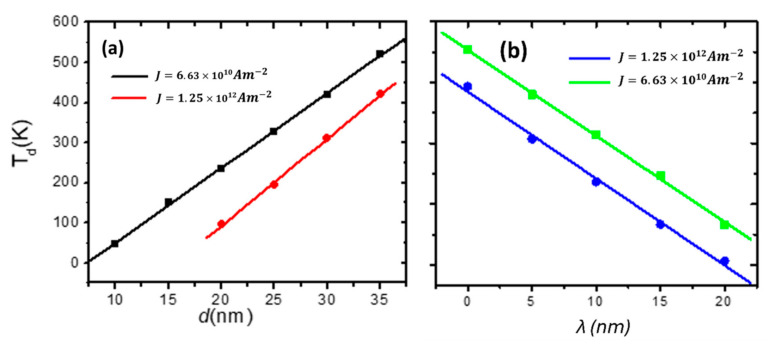
(**a**) Plotting DW depinning temperature (*T_d_*) versus (**a**) *d* and (**b**) *λ* for two values of current density (*J* = 6.63 × 10^10^ Am^−2^ and *J* = 1.25 × 10^12^ Am^−2^). A nanowire with dimensions of 200 × 40 × 3 nm^2^.

Initially, we proceeded to examine the thermal DW stability with respect to the dimensions of the stepped junction (*d* and *λ*). For investigating thermal DW depinning in a stepped nanowire based on *d*, the DW was pinned at the stepped area under a given current density. Then, the device temperature was raised until the DW depinned from the stepped junction. For this investigation, nanowires with *d* values of 10, 15, 20, 25, 30, and 35 nm were used, while *λ* was fixed to 5 nm. Figure 3a shows the relation between DW depinning temperature (*T_d_*) and *d* for two current density values. It shows that *T_d_* has a linear fit with *d* for both current densities. We note that the DW has high thermal stability when increasing *d* and reducing *J*. Moreover, for some values of *d* and *J*, the DW was stable at temperature values higher than room temperature, and this is useful for the applications of DW memory.

Another stepped area dimension that affected DW thermal stability at the stepped area was *λ*. Here, *λ* values were varied by 5 nm, *d* was fixed to 30 nm, and all other parameters were fixed. It was found that the DW thermal stability decreases as *λ* increases. Figure 3b displays the dependence of *T_d_* on *λ*. It was noticed that for *λ* values ≤ 10 nm, the DW needed a device temperature of ≥300 K to move away from the stepped area with a current density of 6.63 × 10^10^ Am^−2^. This means that with small values of λ, the DW has high thermal stability against the temperature at the pinning site. Therefore, low values of *λ* and *J* lead the DW to be more stable with the temperature in the stepped area.

### 3.2. Nanowire Dimensions

**Figure 4:**
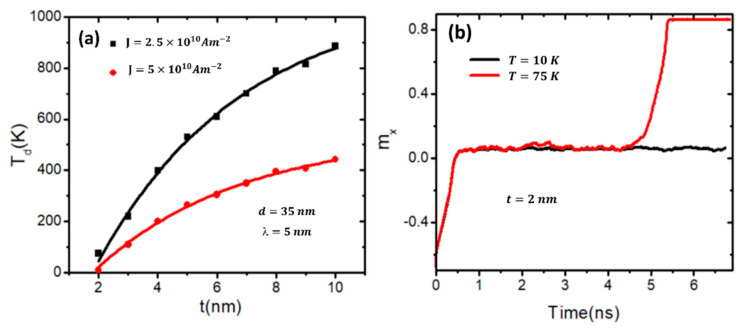
(**a**) A plot of the DW’s thermal depinning (*T_d_*) versus nanowire thickness (*t*) under current density values of 2.5 × 10^10^ Am^−2^ and 5 × 10^10^ Am^−2^. (**b**) A plot of *m_x_* over time for two values of device temperature, 10 K and 75 K, under a current density of 2.5 × 10^10^ Am^−2^. Nanowire with stepped junction of depth and length of 35 nm and 5 nm, respectively.

Then, the effects of the nanowire’s dimensions like the width and the thickness on the DW’s thermal stability were examined. Various thicknesses of devices between 2 and 10 nm were selected in this study, while the length and width of the device stayed at 200 nm and 40 nm, respectively. The stepped area geometry was *d* = 35 nm and *λ* = 5 nm. The magnetic properties were defined as *Ms* = 800 kA/m and *Ku* = 0.5 × 10⁵ Jm^−3^. Figure 4a presents the results of the related effect of the nanowire thickness (*t*) on the DW’s depinning temperature (*T_d_*) for two values of current density: 2.5 × 10^10^ Am^−2^ and 5 × 10^10^ Am^−2^. It was observed that at a current density of 2.5 × 10^10^ Am^−2^, the domain wall (DW) exhibits low thermal stability when the nanowire thickness is ≤3 nm. The DW leaves the stepped area when the device temperature is below room temperature (300 K). However, for thickness values greater than 3 nm, the DW becomes more stable at temperatures higher than room temperature. Conversely, doubling the current density reduces thermal stability. As a result, the DW moves away from the pinning area when the nanowire thickness is less than 6 nm and the temperature is below room temperature. For *t* values of ≥6 nm with both current densities, the DW is stable due to the thermal field, and higher temperature values are needed to depin the DW through the stepped area. Increasing *t* raises the shape anisotropy in the Z-direction, and this helps to reduce the thermal spin fluctuations; the DW stability is increased in the pinning area as a result. For more clarity from these results, a graph of *m_x_* over time was plotted for the DW in a stepped nanowire with a thickness value of 2 nm and two different values of device temperature, 10 K and 75 K, under a driven current density of 2.5 × 10^10^ Am^−2^. It is noted that when the device temperature is 10 K, the DW is stable and pinned at the stepped area, while at a temperature of 75 K, the DW stays stable at the stepped area for around 4.5 ns, then leaves the stepped area and moves to the edge of the nanowire, as shown in Figure 4b.

### 3.3. Magnetic Properties

**Figure 5:**
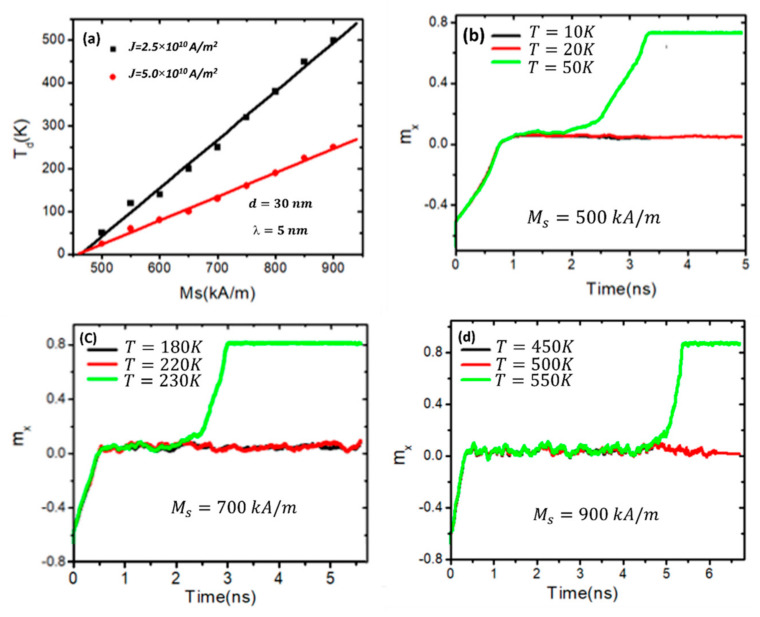
(**a**) A plot of how DW thermal depinning (*T_d_*) varies with *Ms* under current density values of 2.5 × 10^10^ Am^−2^ and ×10^10^ Am^−2^. (**b**) The time dependence of *m_x_* for three temperatures, 10 K, 20 K and 50 K, with an *Ms* of 500 kA/m and under a current density of 2.5 × 10^10^ Am^−2^. (**c**) The change in *m_x_* over time at three temperatures, 180 K, 220 K, and 230 K, a current density of 2.5 × 10^10^ Am^−2^, and *Ms* = 700 kA/m. (**d**) A plot of *m_x_* over time for three temperatures of 450 K, 500 K, and 550 K at an *Ms* of 900 kA/m and 2.5 × 10^10^ Am^−2^. Nanowire with a stepped junction with dimensions of *d* = 35 nm and *λ* = 5 nm.

Magnetic properties such as *Ms* and *K_u_* might also help control the thermal stability of the DW at the pinning sites of the stepped junctions. Thus, the DW’s thermal activation depinning through the stepped area due to magnetic properties was investigated. Figure 5a shows a plot of *T_d_*’s dependence on *Ms* for two different current density values. It can be seen that the trend indicates that the DW’s thermal depinning linearly increases with *Ms*. Additionally, lowering the driven DW current density values is associated with an increase in the DW’s thermal stability.

**Figure 6:**
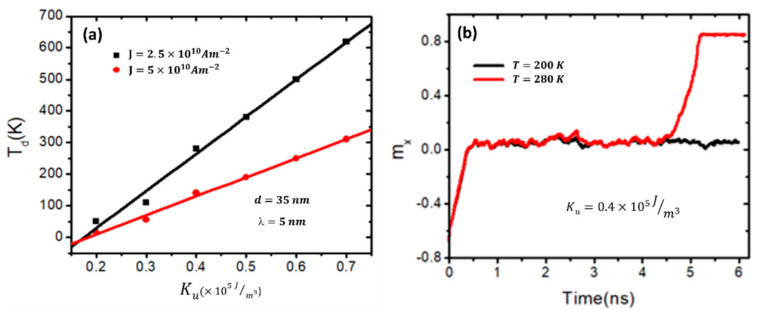
(**a**) A plot of how DW thermal depinning (*T_d_*) varies with *Ms* under current density values of 2.5 × 10^10^ Am^−2^ and ×10^10^ Am^−2^. (**b**) *m_x_* over time for two values of device temperature, 200 K and 280 K, under a current density of 2.5 × 10^10^ Am^−2^.

For a deeper understanding of how increasing *Ms* affects the thermal stability of the DW, Figure 5b illustrates the normalized magnetization (*m_x_*) over time at different device temperatures (10 K, 20 K, and 50 K) with *Ms* fixed at 500 kA/m. From the graph, it is evident that the domain wall (DW) remains pinned at the stepped junction of the nanowire when the device temperature reaches 10 K and 20 K. However, it depins from the stepped area as the temperature increases to 50 K. With an increase in *Ms* to 700 kA/m, the DW moves over the stepped area at a device temperature of 230 K [Figure 5c] and at a temperature of 550 K in the device with an *Ms* of 900 kA/m [Figure 5d]. Furthermore, it is observed that the DW has more oscillations as the device temperature increases, as shown in Figure 5c,d. These thermal magnetization oscillations significantly contribute to an increased occurrence of domain wall (DW) depinning. Moreover, it is worth noting that for values of *Ms* ≥ 700 kA/m, it is seen that the DW is highly stable against the device temperature, which is *T* ≥ 300 K. That means it is stable at higher than room temperature, and this will help maintain the memory lifetime longer. This indicates that its stability extends beyond room temperature, thereby prolonging the lifetime of the memory. Another magnetic parameter that helps to control the thermal stability in the stepped area is magnetic anisotropy (*K_u_*). To investigate the effects of *K_u_* on the DW’s thermal stability in the stepped area, the value of *K_u_* is varied from 0.2 to 0.7 (×10^5^ J/m^3^), while the *Ms* is fixed at 800 kA/m. Figure 6a displays a plot of *T_d_* as a function of *K_u_* for two values of current density. From the plot, it can be seen that *T_d_* increases linearly with *K_u_* for both values of current density. It is seen that for values of *K_u_* ≥ 0.4 × 10^5^ J/m^3^, the DW has stabilized at the stepped area at a device temperature equal to room temperature and higher. For instance, under *J* = 2.5 × 10^10^ Am^−2^ and a *K_u_* value of 0.5 × 10^5^ J/m^3^, the DW leaves the stepped area at a device temperature of 380 K, which is greater than room temperature. These results are essential for constructing racetrack memory with high pinning strength and a long lifetime memory.

These results from adjusting the DW thermal stability at the stepped junction in Figure 6a are confirmed by the plot of *m_x_* over time for two device temperatures (200 K and 280 K) and *K_u_* fixed to 0.4 × 10^5^ J/m^3^ [Figure 6b]. It is seen that the DW in the device at a temperature of 200 K is pinned at the stepped junction. However, in the nanowire at a device temperature of 280 K, the DW travels through the stepped junction to the end of the nanowire.

**Figure 7:**
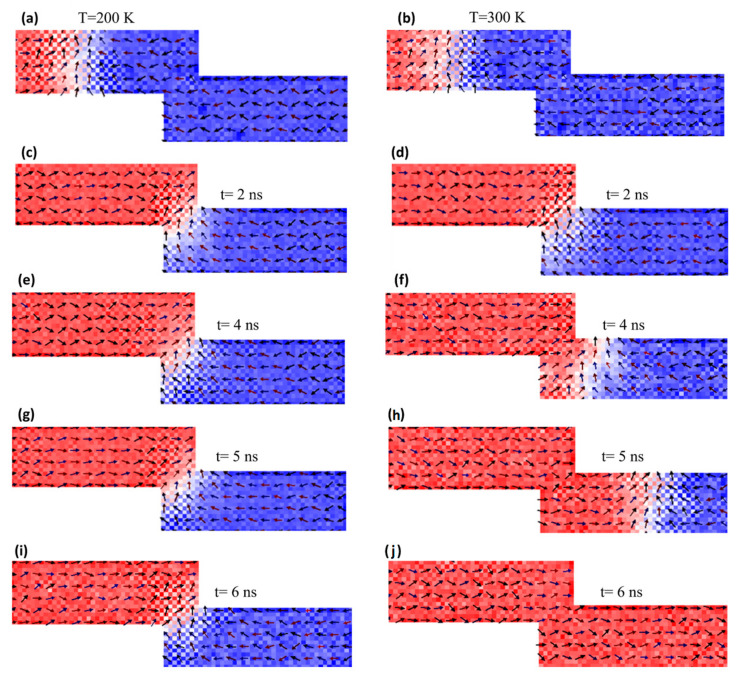
OOMMF images of DW at different positions in the stepped nanowire with constricted area dimensions of *d* = 30 nm and *λ* = 5 nm and two values of device temperature of 200 K (**a**,**c**,**e**,**g**,**i**) and 300 K (**b**,**d**,**f**,**h**,**j**). The magnetic properties are *Ms* = 800 kA/m and *Ku* = 0.4 × 10^5^ J/m^3^.

To clarify the relationship between *K_u_* and the DW’s thermal stability as well as its dynamics, OOMMF images were taken for two different temperature values of 200 K and 300 K. Snapshot images were taken at different times of the DW’s dynamics in a nanodevice with step sizes of *d* = 30 nm and *λ* = 5 nm. The magnetic properties were *Ms* = 800 kA/m and *K_u_* = 0.4 × 10^5^ J/m^3^. A DW was created in the two devices at the two different temperatures under a current density of 2.5 × 10^10^ Am^−2^, as shown in Figure 7a,b. It was observed that the DW took around 1 ns in the nanowire at a temperature of 200 K to reach the stepped junction, while it took 1.5 ns at a temperature of 300 K. It was noted that increasing device temperature increased the DW’s motion in the magnetic nanowires. In the stepped area, it was found that after 6 ns, the DW was still trapped in the pinning area at a temperature of 200 K, as shown in Figure 7c,e,g,i. However, at a nanowire temperature of 300 K, the DW moved away through the stepped junction after 2 ns of reaching the stepped area, as shown in Figure 7f,h, and after 6 ns, the DW vanished at the edge of the nanowire, as shown in Figure 7j.

**Figure 8:**
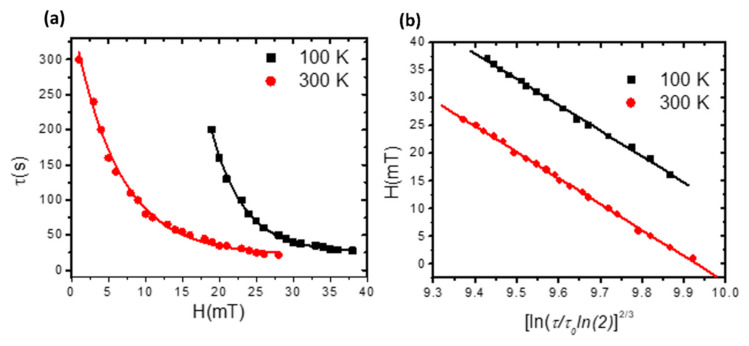
(**a**) Time stability of the DW in the stepped area as a function of the magnetic field for two values of the device temperature. (**b**) The plotting of the magnetic field versus the coefficient lnττ0ln223. A nanowire with dimensions of 200 × 40 × 3 nm^3^ and step dimensions of *d* = 30 nm and *λ* = 10 nm.

Investigating the thermal stability factor and how the DW retention at the pinning area varies with device temperature is crucial for determining the memory lifetime. An examination of the DW’s thermal stability in the stepped area was carried out using a nanowire with *d* = 30 nm and λ = 10 nm step dimensions, a 40 nm width, and a 3 nm thickness. Here, we applied a magnetic field to drive and pin the DW in the stepped area, with the device temperature set to 100 K and 300 K. Magnetization saturation in the negative *x*-direction was first achieved by applying a magnetic field. Next, the magnetic field was reversed to create a DW, which was driven to become pinned in the stepped area, where it stayed for a while before moving away. The DW’s stability time (*τ*) versus the applied magnetic field (*H*) was investigated using the magnetic field values below the depinning field. At temperatures of 100 K and 300 K, the relationship between the DW’s stability time (*τ*) and the applied magnetic field exhibits an exponential decay function, as depicted in Figure 8a. The relation between *τ* and *H* is expressed as follows:

$$\tau =\tau_{0}e^{-\frac{H}{H_{0}}}$$

where $\tau_{0}$ is the inverse of the attempt time (10^−8^ s), and *H*_0_ is the rate of decay.

In order to evaluate the DW’s stability factor in the stepped area, $H\left(t\right)$ was plotted against lnττ0ln223, as shown in Figure 8b, according to Sharrock’s formula [40,41]:

Ht=H0−H0kBTKuV23lnττ0ln223

where $\tau_{0}$ is an attempt time = 10^−8^ s and SF = $\frac{k_{u}V}{K_{B}T}$.

**Figure 9:**
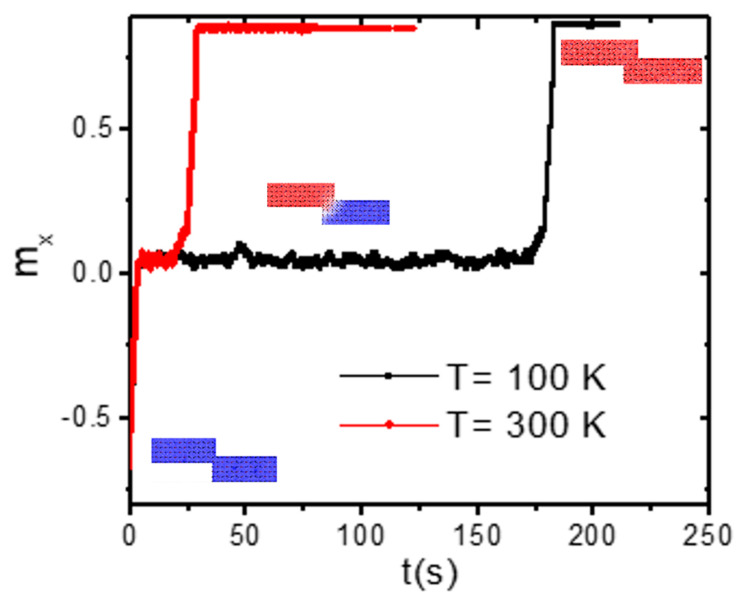
The time-dependent normalized magnetization (*m_x_*) for temperature values of 100 K and 300 K. A nanowire with dimensions of 200 × 40 × 3 nm^3^ and step sizes of *d* = 30 nm and *λ* = 10 nm.

From the graph [Figure 8b], we obtain that the thermal stability factor is 32 at a temperature of 300 K and 33 at 100 K. From the equation, the DW’s staying time in the stepped area was calculated. We found that the DW under 300 K is going to stay for around nine days, while it will remain in the stepped area for 25 days at 100 K. Figure 9 presents the normalized magnetization (*m_x_*) as a function of time for the two temperatures. The dashed area represents the time that the DW remains in the stepped area. It is observed that the DW remains for a longer time at a device temperature of 100 K.

Also, it was seen that the DW obtains high oscillations in the stepped area at a temperature of 300 K. Accordingly, the DW depinning of the stepped area happens rapidly. These results agree with the study of DW oscillations in the stepped area [42].

## 4. Conclusions

In this study, we conclude that the DW’s thermal stability in a stepped area with nanodimensions depends on temperature activation. The micromagnetic simulation shows that the DW’s pinning strength based on the device temperature strongly depends on the nanowire geometries, the pining site dimensions, the magnetic properties, and the current density. A lower current density and narrow and thick nanowires help increase the DW’s thermal stability in the stepped area. In addition, this stability in the stepped area depends on the stepped area dimensions (*d* and *λ*). For some values of *d* and *λ*, the DW is stable under values of temperatures higher than room temperature (300 K), which leads to functional device applications. Furthermore, from the thermal stability factor investigation, we found that under different values of device temperature and nanoscale dimensions, the DW is going to be stable in the stepped area for days. This stability can be increased by increasing *d* and decreasing *λ*.

## Data Availability

All the data used are presented in this paper.
